# A Case of Adenomatous Goiter Involving Diffuse, Acute, and Painful Thyroid Enlargement after Fine-Needle Aspiration Cytology

**DOI:** 10.1155/2014/284912

**Published:** 2014-09-03

**Authors:** Toshiro Shimo, Katsuhiro Tanaka, Ryohei Ogata, Wataru Saito, Yusuke Ohta, Yoshikazu Koike, Tetsumasa Yamashita, Yutaka Yamamoto, Junichi Kurebayashi

**Affiliations:** Department of Breast and Thyroid Surgery, Kawasaki Medical School, 577 Matsushima, Kurashiki, Okayama 701-0192, Japan

## Abstract

The patient was a 44-year-old woman who exhibited a diffuse goiter during health screening. Her medical history did not include any significant medication-based treatment. An echographic examination detected a solid cystic tumor, which measured 21 × 14 × 10 mm, in her right thyroid lobe; however, she displayed normal thyroid function. After fine-needle aspiration cytology had been performed with a 22 G injection needle, the patient immediately complained of compression and pain extending from the front of her neck to her lower chin, which was not accompanied by dyspnea. A second echographic examination revealed diffuse and edematous enlargement and increased internal blood flow in the bilateral thyroid lobes as well as a thyroid nodule. We immediately iced the patient's neck and administered 125 mg methylprednisolone via an intravenous infusion. Within one hour, her symptoms had markedly improved, but acute pain remained. Thus, we continued the steroid (prednisone) treatment, but the dose was gradually reduced from 10 mg/day to 5 mg/day at 1 week after the patient's symptoms disappeared. The mechanism responsible for the patient's condition remains unclear.

## 1. Introduction

Fine-needle aspiration cytology (FNAC) is useful for diagnosing thyroid nodules. Therefore, it is performed as a routine diagnostic method for thyroid tumors. FNAC is generally safe, but various complications such as bleeding can occur in rare cases [1]. We experienced a case of post-FNAC diffuse thyroid enlargement involving acute pain, which seemed to have been caused by an allergic response rather than bleeding.

## 2. Case Report

**Figure 1:**
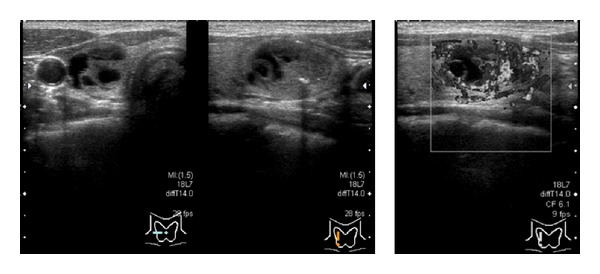
The first echographic examination detected a hypervascular intracystic papillary lesion, which measured 21 × 14 × 10 mm and exhibited high echoic spots, in the right lobe.

**Figure 2:**
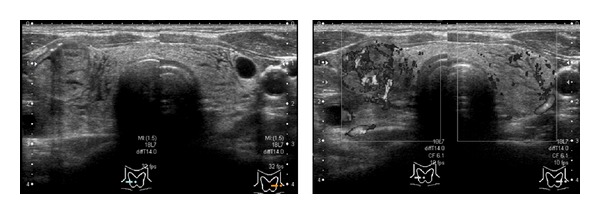
The second echographic examination (performed at symptom onset) showing the bilateral thyroid lobes and a thyroid nodule accompanied by diffuse edematous enlargement and increased internal blood flow.

**Figure 3:**
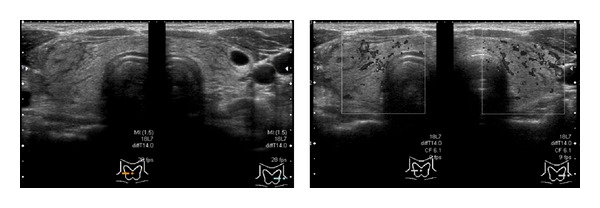
At 1 hour after the appearance of symptoms, the diffuse edematous thyroid enlargement and thyroid nodule had improved.

A 44-year-old Japanese woman visited our hospital complaining of diffuse goiter, which had been detected during health screening. Her medical history did not include any significant medication-based treatment. Palpation demonstrated that her thyroid was slightly enlarged, but no abnormalities were observed in her complete blood count or during biochemical tests. Furthermore, the patient's thyroid function was normal (thyroid-stimulating hormone: 1.18 *μ*IU/mL (0.40–6.00), free thyroxin: 1.16 ng/mL (0.80–1.60)). An echographic examination revealed a solid cystic tumor, which measured 21 × 14 × 10 mm, in her right thyroid lobe. The intracystic tumor appeared as a hypervascular tumor with high echoic spots (Figure 1). The thyroid nodule was subjected to FNAC using a 22 G injection needle. Immediately afterwards, the patient complained of compression and sharp pain extending from the front of her neck to her lower chin, which was not accompanied by dyspnea. We immediately performed a second echographic examination. In addition to the bilateral thyroid lobes and thyroid nodule, diffuse edematous enlargement of the entire thyroid and increased internal blood flow were also observed (Figure 2). Thus, we iced the patient's entire neck and administered 125 mg methylprednisolone via an intravenous infusion. One hour later, the patient's symptoms had rapidly improved, and echographic examinations demonstrated that the diffuse edematous enlargement had also improved (Figure 3). Since some acute pain remained, oral steroid treatment (prednisone) was administered, but the dose was gradually reduced from 10 mg/day to 5 mg/day at 1 week after the patient's symptoms disappeared. The lesion was cytologically diagnosed as benign.

## 3. Discussion

We reported a case involving enlargement of the entire thyroid after FNAC due to edematous changes rather than bleeding. There are few reports about enlargement of the thyroid gland after FNAC in cases that do not involve bleeding. Sumiyoshi and Murakami reported that the incidence of hematoma and diffuse enlargement of the thyroid gland combined with subjective symptoms after FNAC was about 0.03% [1]. To the best of our knowledge, three papers involving nine cases of this condition have been published in Japan [1–3], and five papers involving six cases were published in other countries from 1982 to 2012 [4–8]. Polyzos and Anastasilakis [9] reviewed the latter reports in 2009 and divided the cases into acute transient swelling [4–6] and delayed transient swelling [5, 8]. There is no clear definition of transient swelling. However, cases of acute transient swelling seem to exhibit the following characteristics: (1) sudden onset followed by immediate recovery, (2) the absence of symptoms associated with airway obstruction, and (3) the absence of local symptoms outside of the thyroid. Haas followed up some cases of acute transient swelling using echography and reported that echography is useful for observing the progress of the condition [6]. Delayed transient swelling is defined as swelling that develops from 24 hours after FNAC and does not involve pain [8]. In the present case, steroid treatment was administered, and no thyroid swelling remained two days later.

We performed a literature review of the 10 reported Japanese cases of post-FNAC thyroid enlargement (including our case; Table 1). The patients' ages ranged from 38 to 79 (median age: 58 years) and two males and eight females were included. Three cases involved follicular adenoma, and the remaining seven involved multiple nodular goiters. An 18 G needle was used for the FNAC in one case, whereas 22 G needles were used in the remaining 9 cases. The time between the FNAC and symptom onset ranged from 1 to 3 minutes (min) in two cases and was about 10 min in two patients, 1 hour in one case, 1.5 hours in two cases, and 4 hours in one case. Neck swelling and tenderness were observed in all 10 patients, but dysphagia only occurred in one case. Intravenous steroid treatment was employed in four cases (125 mg methylprednisolone in three cases and 100 mg hydrocortisone in one case). The remaining six cases were simply observed. The patients' symptoms lasted for 1–6 hours in five cases, 1 day in two cases, 1 week in two cases, and 9 days in one case. Regarding the reports from abroad, thyroid enlargement occurred in cases involving thyroid lesions with various histological backgrounds. Nakatake et al. reported the changes in thyroid volume in three cases, in which the thyroid swelled to approximately 2-3 times its normal size [3]. Among the Japanese cases, dysphagia occurred in one case, but dyspnea did not develop in any case. Thus, the clinical characteristics of the Japanese cases accorded with those described in the review by Polyzos and Anastasilakis [9]. Interestingly, in one case transient swelling did not develop after a second FNAC [4].

The cause of diffuse enlargement of the thyroid remains unclear. van den Bruel et al. reported that needle stimulation-induced calcitonin gene-related peptide (CGRP) release caused vasodilation and blood vessel hyperpermeability in a case of medullary carcinoma [5]. In addition, Olesen et al. reported that CGRP localizes in the distal sections of nerves and can induce vasodilation and blood vessel hyperpermeability [10]. Thyroid enlargement has been reported in cases involving lesions of various histological types such as follicular carcinoma, follicular adenoma, and adenomatous goiter [1–8]. In one case, a nerve in the thyroid gland was accidentally stimulated during the FNCA procedure, which resulted in vasodilation, blood vessel hyperpermeability, and general enlargement of the thyroid gland [2]. Sumiyoshi and Murakami assumed that the thyroid enlargement observed in their case had been caused by type I and type IV allergic reactions [1]. Steroid administration is considered to be useful for achieving prompt symptom improvement in cases in which the swelling is caused by blood vessel hyperpermeability or an allergic reaction. However, there have been several cases in which symptoms remained for seven to nine days despite steroid treatment [1–8]. We assume that complex factors are involved in thyroid enlargement. Type I allergic reactions depend on B-cell differentiation and IgE production and histamine release from mast cells induced by IL-4 and IL-13 which helper T cell type II and mast cell produce [11]. Based on the echographic findings with diffuse thyroid edema, time on onset after FNAC, and duration of recovery, we assume that complex factors such as the stimulation of injection and type I allergic reactions induced by unidentified antigen are involved in thyroid enlargement.

Clinicians should carefully follow up patients who undergo FNAC, and patients who display thyroid enlargement involving a rapid onset or severe symptoms, such as dyspnea or acute pain, should be medicated, as steroid treatment was reported to shorten the symptomatic period [1–8].

## 4. Conclusion

We experienced a case of diffuse thyroid enlargement involving acute pain after FNCA in a patient with adenomatous goiter. Also, we observed the echographic changes in the patient's thyroid over time. Such cases are rare, but clinicians must be aware of the condition when performing FNCA. If thyroid enlargement occurs after FNCA, the patient should be carefully followed up, and steroids should be administered where appropriate.

## Figures and Tables

**Table 1:** The 10 cases of post-FNAC thyroid enlargement reported in Japan (including ours).

Report	Age	M/F	Disease	Needle	Time of onset after FNAC	Symptoms	Duration of recovery	Treatment
Sumiyoshi and Murakami [1]	58	F	Follicular adenoma	22G	1.5 hours	Swelling and pain in the neck	1 day	Methylprednisolone (125 mg)
	79	M	Follicular adenoma	22G	10 minutes	Swelling and pain in the neck	1 hour	Course observed
	73	F	Adenomatous goiter	18G	4 hours	Swelling and pain in the neck, dysphagia	1 day	Methylprednisolone (125 mg)
	58	F	Adenomatous goiter	22G	10 minutes	Swelling and pain in the neck	1 week	Course observed
	67	F	Follicular adenoma	22G	10 minutes	Swelling and pain in the neck	6 hours	Course observed
Nimura et al. [2]	38	F	Adenomatous goiter	22G	2-3 minutes	Pressure and pain in the neck	9 days	Hydrocortisone (100 mg)
Nakatake et al. [3]	58	F	Adenomatous goiter	22G	1 hour	Swelling and pain in the neck	A few hours	Course observed
	31	M	Adenomatous goiter	22G	1.5 hours	Acute neck pain, no swelling	A few hours	Course observed
	45	F	Adenomatous goiter	22G	2 hours	Swollen neck	A few hours	Course observed
Our case	44	F	Adenomatous goiter	22G	1-2 minutes	Pressure and pain in the neck	1 week	Methylprednisolone (125 mg)
